# Vagus nerve plays a pivotal role in CD4+ T cell differentiation during CVB3-induced murine acute myocarditis

**DOI:** 10.1080/21505594.2020.1869384

**Published:** 2021-01-15

**Authors:** Li Yue-Chun, Xiao-Hong Gu, Ge Li-Sha, De-Pu Zhou, Chao Xing, Xiao-Ling Guo, Lu-Lu Pan, Shi-Yang Song, Li-Li Yu, Guang-Yi Chen, Jia-Feng Lin, Mao-Ping Chu

**Affiliations:** a Institute of Cardiovascular Development and Translational Medicine, The Second Affiliated Hospital and Yuying Children’s Hospital of Wenzhou Medical University, Wenzhou, China; b Department of Pediatric Emergency, The Second Affiliated Hospital and Yuying Children’s Hospital of Wenzhou Medical University, Wenzhou, China; c Department of Clinical Laboratory, The Second Affiliated Hospital and Yuying Children’s Hospital of Wenzhou Medical University, Wenzhou, China; d Center of Scientific Research, The Second Affiliated Hospital and Yuying Children’s Hospital of Wenzhou Medical University, Wenzhou, China; e Child Health Manage Department, Maternal and Child Health Care Institution, Wenzhou, China

**Keywords:** Viral myocarditis, vagus nerve, cholinergic anti-inflammatory pathway, CD4^+^ T cells

## Abstract

Abnormalities in CD4^+^ T cell (Th cell) differentiation play an important role in the pathogenesis of viral myocarditis (VMC). Our previous studies demonstrated that activation of the cholinergic anti-inflammatory pathway (CAP) alleviated the inflammatory response. In addition, we observed that right cervical vagotomy aggravates VMC by inhibiting CAP. However, the vagus nerve’s effect on differentiation of CD4^+^ T cells has not been studied in VMC mice to date. In this study, we investigated the effects of cervical vagotomy and the α7nAChR agonist pnu282987 on CD4^+^ T cell differentiation in a murine myocarditis model (BALB/c) infected with coxsackievirus B3 (CVB3). Splenic CD4^+^ T cells from CVB3-induced mice obtained and cultured to investigate the potential mechanism of CD4^+^ T cell differentiation. Each Th cell subset was analyzed by flow cytometry. Our results showed that right cervical vagotomy increased proportions of Th1 and Th17 cells and decreased proportions of Th2 and Treg cells in the spleen. Vagotomy-induced upregulation of T-bet, Ror-γ, IFN-γ, and IL-17 expression while downregulating the expression of Gata3, Foxp3, and IL-4 in the heart. In addition, we observed upregulated levels of proinflammatory cytokines, aggravated myocardial lesions and cellular infiltration, and worsened cardiac function in VMC mice. Pnu282987 administration reversed these outcomes. Furthermore, vagotomy inhibited JAK2-STAT3 activation and enhanced NF-κB activation in splenic CD4^+^ T cells. The CD4^+^ T cell differentiation was related to JAK2-STAT3 and NF-κB signal pathways. In conclusion, vagus nerve modulates the inflammatory response by regulating CD4^+^ T cell differentiation in response to VMC.

## Introduction

Viral myocarditis (VMC) can produce nonspecific interstitial myocardial inflammation and necrosis, leading to malignant arrhythmias, heart failure, and even sudden cardiac death in young patients [1]. The development of VMC can be divided into three phases. First, the entry of the virus into myocytes causes myocardial damage and induces the host’s innate immune system to remove the pathogens. The second phase is characterized by adaptive immune and autoimmune responses. In this stage, activation of T cells, cytokines, and antibodies begin to target on the normal organs, which aggravates cardiac damage and impairs contractile function. In the third phase, persist autoimmune processes in the myocardium can cause myocardial remodeling and lead to the development of dilated cardiomyopathy [2,3]. The current researches on VMC are mainly focused on the last two phases. CD4⁺ T cells, also known as Th cells, are recognized as playing crucial roles in mediating the immune response in these phases [4,5]. Some studies have reported inflammatory responses are significantly reduced in CD4 knockout mice with myocarditis [6]. Recently, subsets of Th cells, including Th1, Th2, Th17, and Treg cells, have been reported to exhibit differential, or even opposing, effects on the pathogenesis of myocarditis [4]. Huber et al. discovered that male mice were more susceptible to developing VMC from coxsackievirus B3 (CVB3) infection than were female mice [7]. Immune response to CVB3 is dominated by Th1 cell-mediated immunity in male mice, while VMC immune mediation occurs via Th2 in female mice. Interestingly, the development of myocarditis was significantly inhibited in male VMC mice that were transplanted with Th2 cells from female mice [7]. Fairwearher and colleagues reported that Th1 responses increased acute inflammation, yet this response protected against CVB3 myocarditis by inhibiting viral replication and Th2 responses. Th2 responses reduced CVB3 myocarditis by inhibiting inflammation, but can promote progression to dilated cardiomyopathy by stimulating cardiac remodeling [8]. Many studies have shown that Th17 cells and their cytokines promote the development of myocarditis [8,9]. In contrast, Treg cells have powerful anti-inflammatory effects on myocarditis, and maintaining the balance between Th17 and Treg cells is crucial in murine and human myocarditis [10,11]. In distinct myocarditis models, the peak time point of acute myocarditis was different, the CVB3-only model was d 7, the hybrid-CVB3 model was d 10, and the experimental autoimmune myocarditis was d 21 [8]. In VMC pathogenesis, multiple cytokines are activated that are closely related to those seen during differentiation of the Th cell subsets.

Recently, anti-inflammatory mechanisms mediated by the nervous system have generated considerable interest. Autonomic nervous system dysfunction with sympathetic activation and vagus withdrawal contribute to the progression of VMC [12]. Our previous studies indicated that excessive sympathetic activation upregulates levels of inflammatory cytokines that aggravate inflammatory lesions, whereas carvedilol, the nonselective β-blocker, alleviated the inflammatory response [13–15]. The vagus nerve, involved in control of heart rate and in hormone secretion, is also an immunomodulator. The cholinergic anti-inflammatory pathway (CAP) is mediated by the vagus nerve, which is the longest cranial nerve that dominates most of peripheral organs and relies on the α7-nicotinc acetylcholine receptor (α7nAChR) to alleviate the inflammatory response and to repair immune injury from VMC [16]. Signal transmission and activation of CAP is dependent upon the vagus nerve, and α7nAChR bond with ligands to regulate the signaling pathway and modify inflammation. Activating the efferent fibers of the vagus nerve regulates systemic and local inflammatory responses via CAP [17]. Our previous studies demonstrated that activation of CAP with the α7nAChR agonist nicotine alleviates the inflammatory response [18,19] and right cervical vagotomy aggravates VMC by inhibiting CAP [20]. However, the effect of the vagus nerve on differentiation of CD4^+^ T cells has not been studied in VMC mice. Therefore, in this study, we examined the potential effects of the vagus nerve on differentiation of CD4^+^ T cell subsets. Furthermore, we investigated the effects of cervical vagotomy in a murine model of myocarditis infected with CVB3. Finally, we examined the underlying mechanism of Th cell differentiation after activation or inhibition of α7nAChR in CD4^+^ T cells.

## Materials and methods

### Animal preparation

This study conformed to the Guide for the Care and Use of Laboratory Animals by the National Institutes of Health (NIH Publication, 8th Edition, 2011) and China Animal Welfare Law. The Wenzhou Medical University Committee on Ethics in the Use and Care of Laboratory Animals approved conduct of this study. Male BALB/c mice (SPF class, 20–24 g, 4 to 6 weeks) were purchased from Shanghai Laboratory Animal Center, China. All mice were maintained in the Wenzhou Medical University animal facilities under specific pathogen-free conditions. All experimental animals were sacrificed with an overdose of pentobarbital (100 mg/kg, one dose intraperitoneally).

### Viral myocarditis

CVB3 (Nancy strain, USA) was maintained and expanded using Hep2 cells. The viral titer was determined by 50% tissue culture infectious dose (TCID50) assay. Mice were randomly assigned to six groups receiving the following treatments: normal mice (Control), VMC mice (VMC), VMC mice with unilateral (right-sided) cervical vagus nerve dissociation without mutilation of cervical vagus nerve (VMC+Sham), VMC mice with vagotomy (VMC+Vag), VMC mice with vagotomy and pnu282987 administration (VMC+V+P), and VMC mice with pnu282987 administration (VMC+Pnu). In preparation for vagotomy, mice were injected intraperitoneally with 2% sodium pentobarbital (40 mg/kg) and fixed in place on the operating table. After shaving and sterilizing the neck and chest, a one-centimeter incision was made in the middle of the neck, and the tissue was separated. The right cervical vagus nerve was dissociated and cut off, and the incision was sutured. In the VMC+Sham group, the vagus nerve was only isolated from surrounding tissue but was not transected. All operations were performed under anesthesia. After surgery 24 h, all mice were intraperitoneally inoculated with 0.1 mL of normal saline containing 10^5^ TCID50 of CVB3 except for the Control group. The Control group was intraperitoneally injected with 0.1 ml saline solution. The day of virus inoculation was defined as d 0. For agonist treatment, mice received intraperitoneal injection of pnu282987 (Pnu, a selective α7nAChR agonist, Sigma, USA, 2 mg/kg/d, 0.1% DMSO) from d 1 to 14 [21], while controls were injected with the same dose of vehicle (0.1% DMSO in saline).

### Doppler echocardiography study

Transthoracic echocardiography with an M-mode transducer (12-MHz phased-array transducer; Sonos 5500, Philips USA, Bothell, WA) was performed by an experienced technician who was blinded to study groups on d 7 and 14 [15]. After anesthesia, the chest was shaved and mice were placed on a heating pad. At the papillary muscle level, short-axis views of M-mode tracings were recorded through the anterior and posterior left ventricular walls to measure LV end-diastolic dimension (LVEDd), LV end-systolic dimension (LVESd), and LV fraction shortening (LVFS). LV ejection fraction (LVEF) values were obtained according to the Simpson approach.

### Myocardial histopathology

Heart tissue was embedded in paraffin. Samples were then sectioned into 5-μm-thick slices and subjected to hematoxylin and eosin (H&E) staining. Each heart was cut into an average of three slices, and each slice was used to observe three visual fields. Two independent observers blind to the experimental conditions assigned pathological scores based on the following semi-quantitative scale: 0 = no lesion; 1 = lesion involving <25% of the myocardium; 2 = lesions involving 25% to 50%; 3 = lesions involving 50% to 75%; and 4 = lesions involving 75% to 100%, as previously described [22]. The scores for every section were averaged.

### Western blot analysis

The total protein from heart tissue and splenic CD4^+^ T cells was lysed in Radio Immunoprecipitation Assay (RIPA) Lysis Buffer (ThermoFisher, 89,900), with protease inhibitors (Beyotime, Jiangsu, China, ST506-2) and phosphatase inhibitors (Applygen Technologies, Beijing, China, P1260) for 30 min under rotating agitation. The splenic CD4^+^ T cells were purified by magnetic-activated cell sorting (MACS, Miltenyi Biotec, Germany). A BCA protein assay kit (ThermoFisher, 23,235) was used to measure protein concentration. Then, 40 μg heart tissue protein or 10–20 μg CD4^+^ T cell protein were mixed with one-fifth volume of 5× loading buffer. The mixture was denatured by boiling at 100°C for 10 min. Samples were separated with 8% to 12% SDS-PAGE and transferred to a polyvinylidene difluoride (PVDF) membrane. Membranes were blocked in 5% nonfat milk at room temperature for 2 h. After incubation with primary antibody at 4°C overnight and secondary antibodies, membranes were detected with the enhanced chemiluminescence detection system (Millipore, Billerica, MA). The antibodies used included the following: rabbit anti-IL-1β monoclonal antibody (1:1000; Cell Signaling Technology, #12,426), rabbit anti-IL-6 monoclonal antibody (1:1000; Cell Signaling Technology, #12,912), rabbit anti-TNF-α monoclonal antibody (1:1000; Cell Signaling Technology, #11,948), rabbit anti-T-bet polyclonal antibody (1:1000, Abcam, #ab53174), rabbit anti-GATA3 polyclonal antibody (1:1000; Abcam, #ab106625), mouse anti-Ror-γ monoclonal antibody (1:1000; R&D System Technology, #600,214), rabbit anti-Foxp3 polyclonal antibody (1:1000; Abcam, # ab54501), rabbit anti-IFN-γ polyclonal antibody (1:1000; Abcam, #ab216642), rat anti-IL-4 monoclonal antibody (1:1000; Abcam, #ab11524), rabbit anti-IL-17 polyclonal antibody (1:1000; Abcam, #ab79056), rabbit anti-NF-κB p65 monoclonal antibody (1:1000; Cell Signaling Technology, #8242), rabbit anti-phospho-NF-κB p65 (Ser536) monoclonal antibody (1:1000; Cell Signaling Technology, #3033), rabbit anti-IκB-α monoclonal antibody (1:1000; Cell Signaling Technology, #4812), rabbit anti-phospho-IκBα (Ser32) monoclonal antibody (1:1000; Cell Signaling Technology, #2859), rabbit anti-JAK2 monoclonal antibody (1:1000; Cell Signaling Technology, #3230), rabbit anti-phospho-JAK2 monoclonal antibody (1:1000; Cell Signaling Technology, #4406), rabbit anti-STAT3 monoclonal antibody (1:1000; Cell Signaling Technology, #12,640), rabbit anti-phospho-STAT3 monoclonal antibody (1:1000; Cell Signaling Technology, #9145), and rabbit anti-GAPDH polyclonal antibody (1:1000; Cell Signaling Technology, #5174). The experiment was repeated 3 times for each sample.

### Quantitative real-time PCR (qPCR)

Total RNA, extracted with TRIzol Reagent (Invitrogen, USA), was converted into cDNA with a ReverTra Ace qPCR RT Kit (TOYOBO, JAPAN) according to manufacturer’s instructions. Real-time polymerase chain reaction (qPCR) was performed with a BIO-RAD® Real-Time Detection System (Bio-rad, USA) using iQ SYBR Green Supermix (Bio-rad, USA). Calculation of mRNA expression levels was performed using the $2^{\left(-\Delta \Delta CT\right)}$ method and normalized against GAPDH levels. All reactions were run in triplicate. The primer sequences are listed in **Supplemental material (Table 1. Primer sequences used for molecular analysis).**

### Flow cytometry analysis

Mouse spleens were aseptically removed from animals in each group, washed, and ground in phosphate buffer solution (PBS), and filtered to form single-cell suspensions. Erythrocytes were subsequently removed using a red blood cell lysis buffer. Then, cell suspension was centrifugated for 5 min at 300 g. Supernatant was discarded and the cell pellet was resuspended. After cell count and viability check cells were centrifugated. Cells were resuspended in RPMI 1640 containing 10% fetal bovine serum at a density of 1 × 10⁶ cells/mL. To analyze the percentages of Th1, Th2, and Th17 cells, cultures were stimulated for 6 h with leukocyte activation cocktail (BD Pharmingen, USA, #550,583) at 5% CO2, 37°C in a 24-well culture plate. Cells were harvested and stained with FITC-conjugated anti-mouse CD4 antibody (BD Pharmingen, USA, #553,046). After washing, fixing, and permeabilizing, cells were stained with PerCP-Cyanine5.5-conjugated anti-mouse IFN-γ antibody (BD Pharmingen, USA, #560,660), APC-conjugated anti-mouse IL-4 antibody (BD Pharmingen, USA, #554,436), and PE-conjugated anti-mouse/rat IL-17A antibody (BD Pharmingen, USA, #560,436). For Treg cells, suspended unstimulated CD4^+^ T cells were stained with FITC-conjugated anti-mouse CD4 antibody, APC-conjugated anti-mouse CD25 antibody (BD Pharmingen, USA, #561,048), and PE-conjugated anti-mouse/rat Foxp3 antibody (BD Pharmingen, USA, #560,414). Isotype controls were utilized to enable correct compensation and confirm antibody specificity. The isotype controls we used are as followed: PerCP-Cy™5.5 Rat IgG1, κ Isotype Control (BD Pharmingen, USA, #554,436), PE Rat IgG1, κ Isotype Control (BD Pharmingen, USA, #554,685), and APC Rat IgG1, κ Isotype Control (BD Pharmingen, USA, #554,686). Cells were measured on a BD FACS-Canto^TM^ II flow cytometer, and data were analyzed with FlowJo software.

### *CD4^+^ T cell culture* in vitro

MACS was used to select CD4^+^ T cells positively from the spleen of CVB3-induced myocarditis mice on d 7, according to manufacturer’s instructions. The day of cells separation was defined as d 0. Cells were cultured in twelve-well plates and randomly divided into seven groups: (1) PBS group, (2) Pnu group (30 μM), (3) Pnu (30 μM) + pyrrolidinedithiocarbamate (PDTC, a selective NF-κB inhibitor, Sigma, USA, 50 μM) group, (4) Pnu (30 μM) + nsc74859 (NSC, a STAT3 inhibitor, MedChem Express, USA, 100 μM) group, (5) methyllycaconitine (MLA, a selective α7nAChR antagonist, Sigma, USA, 10 μM) group, (6) MLA (10 μM) + PDTC (50 μM) group, (7) MLA (10 μM) + NSC (100 μM) group. All drugs were administered once daily on d 1 to 5. Finally, cells were harvested and used for Western blot and flow cytometry analyses.

### Statistical analysis

Data are expressed as mean ± SD. Outcomes were compared among groups using one-way analysis of variance (ANOVA) followed by Dunnett’s multiple comparison test. Categories variables were performed by rank-sum test. Kruskal–Wallis test followed by multiple comparison test was used to assess differences between myocardial pathological scores. Statistical analyses were performed using SPSS 22.0 software. A value of *p*< 0.05 was considered statistically significant.

## Results

### Echocardiographic findings

**Figure 1.**
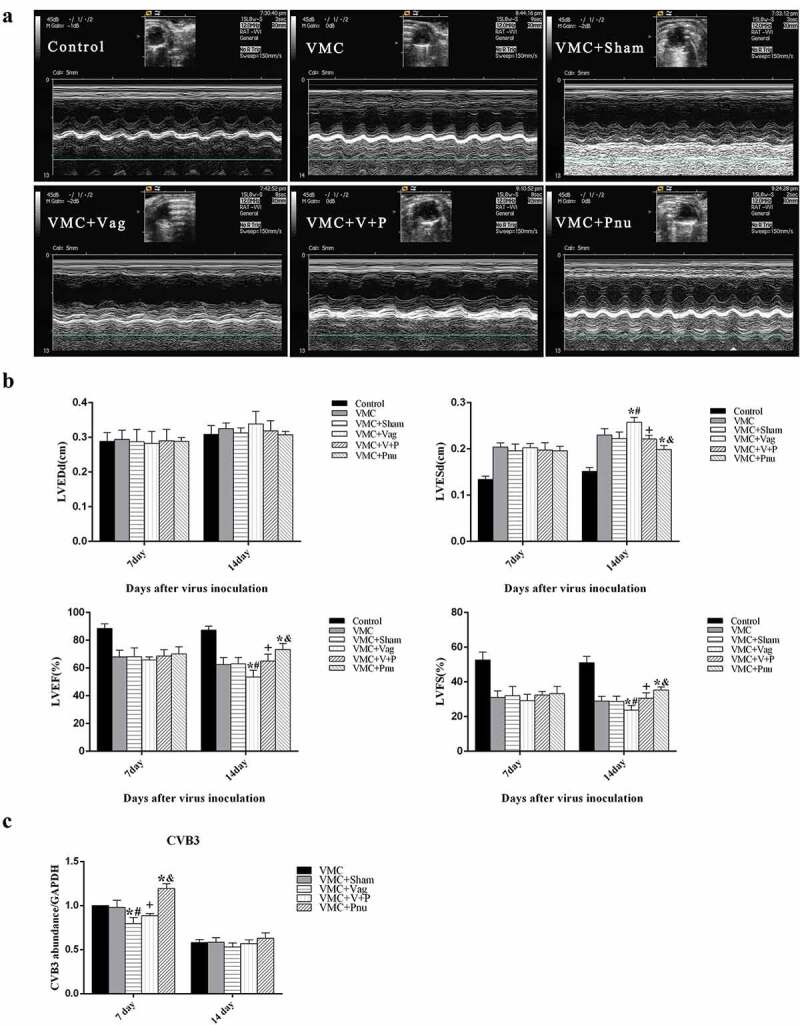
The echocardiography results from the six experimental groups (*n* = 8 per group) and Viral replication in the myocardium (*n* = 5 per group). (a), Typical M-mode echocardiograms of the short-axis midventricular view on d 14. (b), The differences of LVEDd, LVESd, LVEF, and LVFS among six groups on d 7 and 14. (c) Viral replication in the myocardium of each group. Data are expressed as means ± SD. *p< 0.05 vs. VMC group. #*p*< 0.05 vs. VMC+Sham group. +*p*< 0.05 vs. VMC+Vag group. &*p*< 0.05 vs. VMC+V+P group

After vagotomy, all mice were infected with CVB3 except for the Control group. pnu282987 was given to VMC mice for consecutive 14 d, starting 24 h after viral inoculation. On d 7 and 14, echocardiography was performed on more than 8 mice in each group. As shown in Figure 1a, M-mode images from VMC, VMC+Sham, VMC+Vag, and VMC+V+P groups showed that left ventricular movements were weaker than that in control and VMC+Pnu groups. LVEF and FS of VMC, VMC+Sham, VMC+Vag, VMC+V+P, and VMC+Pnu groups were significantly decreased compared to the control group, but LVESd was slightly elevated (Figure 1b). By d 14, mice that had undergone vagotomy exhibited decreased LVEF and FS, but increased LVESd in the VMC+Vag group compared with the VMC group (*p*< 0.05). The damage from vagotomy in the VMC+Vag group was attenuated when pnu282987 was administered to that group (*p*< 0.05). And pnu282987 administration significantly improved LVEF and LVFS, but reduced LVESd in the VMC+Pnu group compared to the VMC group (*p*< 0.05). LVEDd, LVESd, LVEF, and FS measurements were similar between VMC and VMC+Sham groups (*p*> 0.05).

### Viral replication in the heart

We detected the levels of CVB3-RNA abundance in the infected heart tissues. On d 7, Vagotomy decreased the CVB3-RNA abundance in the VMC+Vag group compared to VMC and VMC+Sham groups (*p*< 0.05). Pnu282987 administration increased CVB3 replication in VMC+Pnu group (*p*< 0.05). On d 14, there were no significant differences among all groups. Additionally, the level of viral replication in the myocardium was decreased on d 14 compared with d 7 (Figure 1c).

### Myocardial histopathology

**Figure 2.**
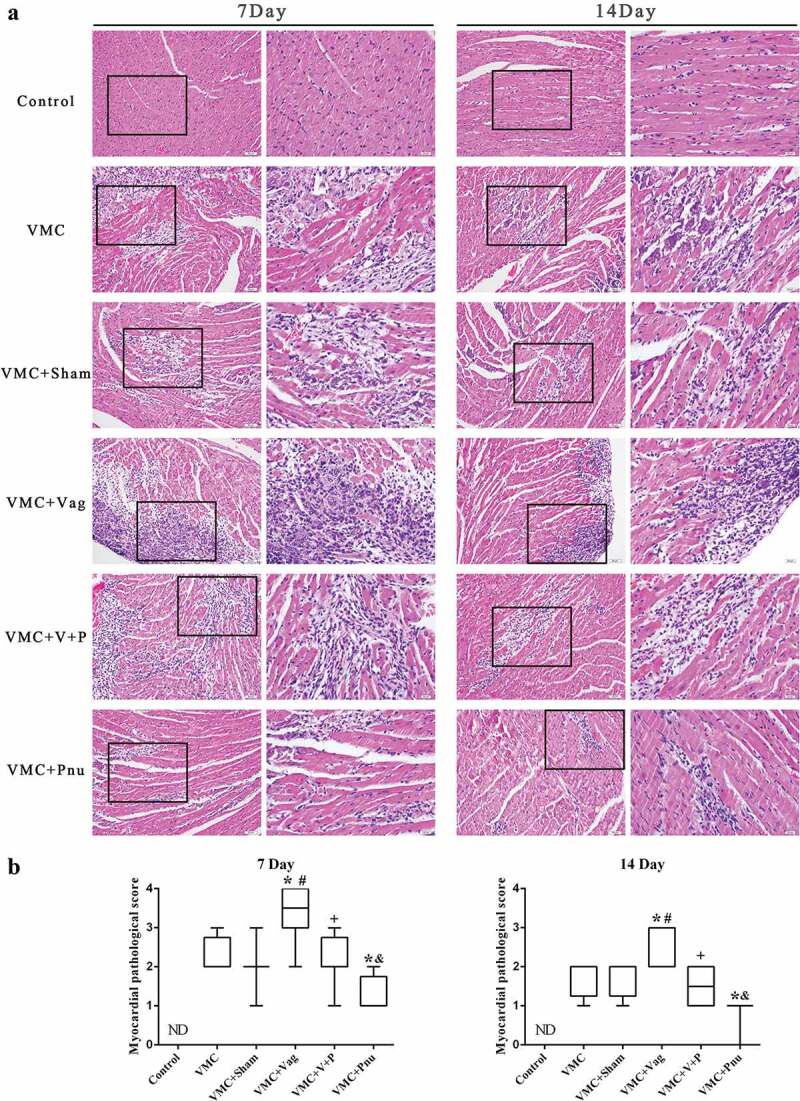
Comparison of myocardial histopathological changes among the six groups on d 7 and 14 (*n* = 8 per group). (a) Typical histopathological changes for each group. (left magnification × 200, scale bar = 50 μm; right magnification × 400, scale bar = 20 μm). (b) Box-plots of the histopathology scores for each group. Data are expressed as means ± SD. ND, not detected. *p< 0.05 vs. VMC group. #*p*< 0.05 vs. VMC+Sham group. +*p*< 0.05 vs. VMC+Vag group. &*p*< 0.05 vs. VMC+V+P group

We examined changes in myocardial histopathology by H&E staining (Figure 2a). On both d 7 and 14, there were no evident pathological changes in the control group, while VMC and VMC+Sham groups showed severe injuries accompanied by cellular infiltration and necrosis. Vagotomy exacerbated the extent of cellular infiltration and necrosis in VMC mice (*p*< 0.05). The pathological damage was significantly reduced in VMC+Vag mice with pnu282987 treatment (*p*< 0.05). Likewise, pnu282987 administration in VMC mice exhibited smaller and more limited foci of cellular infiltration and necrosis (*p*< 0.05). (Figure 2b)

### Expression of proinflammatory cytokines in the heart on d 7 and 14

**Figure 3.**
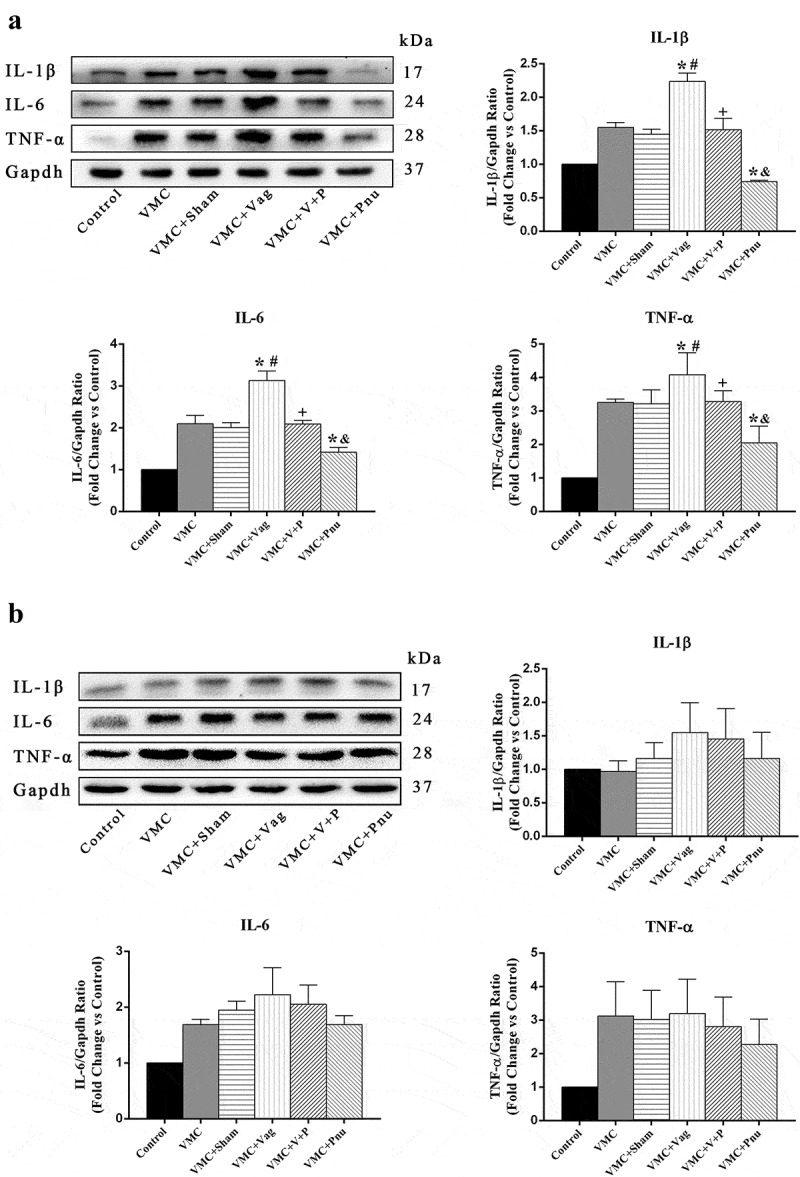
Expression of proinflammatory cytokines by Western blot analysis in myocardial tissue on d 7 and 14 (*n* = 6 in each group). (a) and (b) are the absolute intensity ratio of proinflammatory cytokines on d 7 and 14, respectively. On d 7, VMC mice with vagotomy exhibited significantly higher expression of IL-1β, IL-6, and TNF-α. Whereas pnu282987 administration decreased the expression of proinflammatory cytokines in VMC and VMC+Vag mice. No obvious differences were observed in the expression of proinflammatory cytokines across the five myocarditis groups on d 14. Data are expressed as means ± SD. *p< 0.05 vs. VMC group. #*p*< 0.05 vs. VMC+Sham group. +*p*< 0.05 vs. VMC+Vag group. &*p*< 0.05 vs. VMC+V+P group

To evaluate the effects of the vagus nerve on proinflammatory cytokines in the heart, we measured expression of IL-6, IL-1β, and TNF-α by Western blot. As shown in Figure 3, on d 7, the levels of IL-1β, IL-6, and TNF-α were significantly increased by 1.54-, 1.87-, and 3.07-fold in VMC group and 1.46-, 1.83-, and 3.03-fold in VMC+Sham group compared to Control group, respectively. Vagotomy upregulated the release of IL-1β, IL-6, and TNF-α by 1.43-, 1.28-, and 1.40-fold in VMC+Vag group compared to VMC group (*p*< 0.05). However, pnu282987 administration reduced proinflammatory cytokine production by 0.68-, 0.67-, and 0.53-fold in VMC mice and 0.68-, 0.74-, and 0.70-fold in VMC+Vag mice, respectively (*p*< 0.05). There were no significant differences of IL-1β, IL-6, and TNF-α expression levels among the five myocarditis groups on d 14.

### Levels of specific transcription factors and cytokines in Th cell subsets from the heart on d 7 and 14

**Figure 4.**
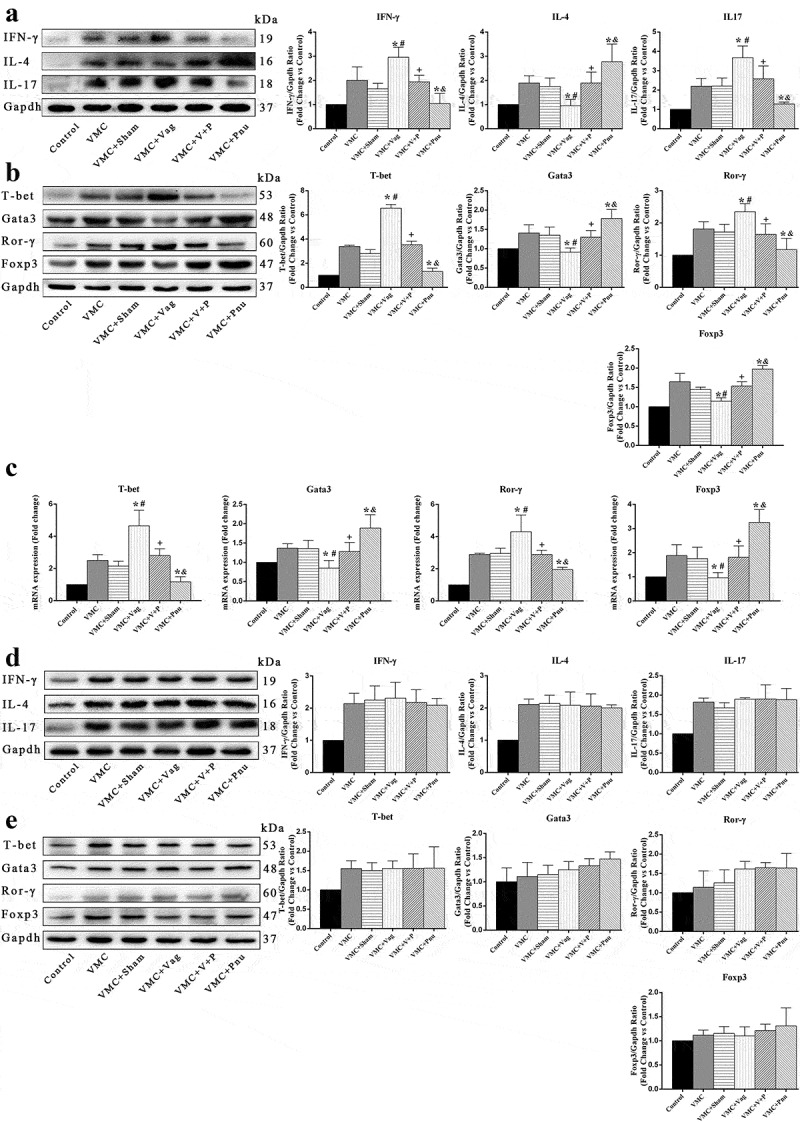
Expression of specific transcription factors and cytokines for Th cell subsets in the heart on d 7 and 14 (*n* = 6 per group). (a) and (b) denote protein expression of the cytokines of Th cell subsets by Western blot analysis. (c) and (d) denote protein expression of the specific transcription factors of Th cell subsets by Western blot analysis. C. Expression levels of specific transcription factors mRNA for Th cell subsets by qPCR analysis. Data are expressed as means ± SD. *p< 0.05 vs. VMC group. #*p*< 0.05 vs. VMC+Sham group. +*p*< 0.05 vs. VMC+Vag group. &*p*< 0.05 vs. VMC+V+P group

T-bet, Gata3, Ror-γ, and Foxp3 are specific transcription factors found in Th1, Th2, Th17, and Treg cells, respectively. IFN-γ, IL-4, and IL-17 are specific cytokines for Th1, Th2, and Th17 cells, respectively [23]. Hence, we observed the proportion variations on Th1, Th2, Th17, and Treg cells with changes in expressions of specific transcription factors and cytokines. As shown in Figure 4a and b, blocking CAP with vagotomy significantly reduced protein expression of Th2 cell-associated Gata3 and IL-4 as well as Treg cell-associated Foxp3 in the heart and elevated levels of Th17 cell-related Ror-γ and IL-17 as well as Th1 cell-related T-bet and IFN-γ in VMC mice on d 7 (*p*< 0.05). In contrast, pnu282987 treatment caused the opposite effect, increasing expression of Gata3, Foxp3, and IL-4 and decreasing expression of T-bet, Ror-γ, IFN-γ, and IL-17 in VMC+Pnu and VMC+V+P groups compared with VMC and VMC+Vag groups, respectively (*p*< 0.05). The mRNA levels of Th cells specific transcription factors were also detected, and the results were consistent with results of the Western blot analyses (Figure 4c). On d 14, there were no obvious differences of specific transcription factor and cytokine expressions for Th cell subsets among the five myocarditis groups (Figure 4d and e).

### *Th subset percentages in the spleen* in vivo

**Figure 5.**
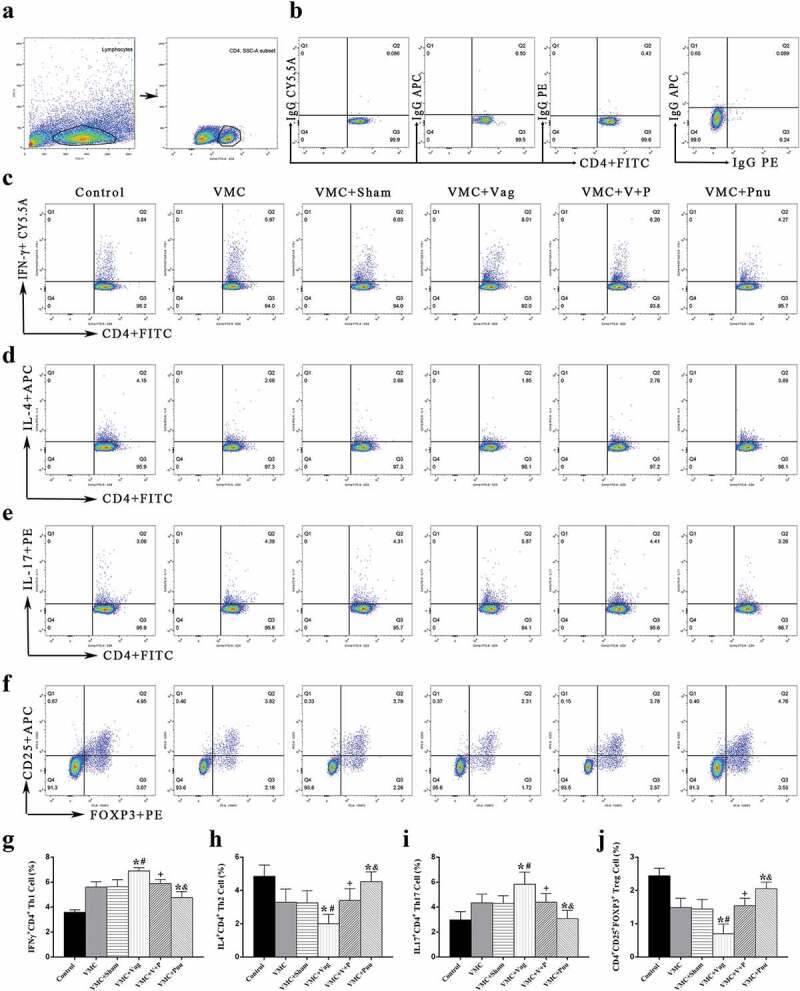
Th subsets by flow cytometry analysis in the spleen on d 7 (*n* = 6 per group). (a), CD4^+^ T cells were identified from total lymphocyte populations based on forward scatter and side scatter. (b), Isotype controls for Th1, Th2, Th17, and Treg cells. (c) through (f), The representative results for each group. (g)through (j), The percentage of double-positive cells for each group. CD4, IFN-γ, IL-4, IL-17, CD25, and FOXP3 were respectively labeled by FITC, PE-CY5.5, APC, PE, APC, and PE. Data are expressed as means ± SD. *p< 0.05 vs. VMC group. #*p*< 0.05 vs. VMC+Sham group. +*p*< 0.05 vs. VMC+Vag group. &*p*< 0.05 vs. VMC+V+P group

Flow cytometry was used to detect the percentage of CD4^+^ T cell subsets in the spleen. On d 7 post-vagotomy, the percentages of IFN-γ^+^ CD4^+^ Th1 and IL-17^+^ CD4^+^ Th17 cells were increased, but the percentages of IL-4^+^ CD4^+^ Th2 and CD4^+^ CD25^+^ Foxp3^+^ Treg cells in VMC mice were decreased (*p*< 0.05). In contrast, the percentages of Th1 and Th17 cells were down regulated, and the percentages of Th2 and Treg cells were up regulated when pnu282987 were administered in VMC mice (*p*< 0.05). Furthermore, pnu282987 partly mitigated the percent variations of Th subsets in VMC+Vag mice (*p*< 0.05) (Figure 5).

### *Protein expressions of JAK2-STAT3 and NF-κB signal pathways in splenic CD4^+^ T cells* in vivo

**Figure 6.**
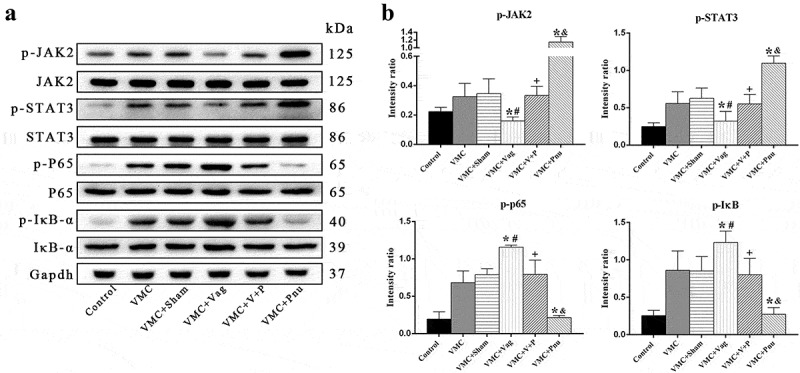
Western blot analysis demonstrating protein expression of JAK2-STAT3 and NF-κB pathways in splenic CD4^**+**^ T cells on d 7 ***in vivo*** (*n* = 6 in each group). (a), Representative protein expression of signaling pathways. (b), The quantitative analysis of p-JAK2, p-STAT3, p-P65, and p-IκB-α expression for each group. Data are expressed as means ± SD. *p< 0.05 vs. VMC group. #*p*< 0.05 vs. VMC+Sham group. +*p*< 0.05 vs. VMC+Vag group. &*p*< 0.05 vs. VMC+V+P group

Based on above results, inhibiting or activating CAP by vagotomy or pnu282987 regulates differentiation of Th cell subsets, but the underlying mechanisms whereby this occurs are unknown. The NF-κB signaling pathway is critical for the activation of immune cells and formation of inflammatory cytokines [24]. The JAK-STAT signal pathway is stimulated by mediators that play a key role in cell proliferation, differentiation, migration, and apoptosis [25]. Therefore, we detected the protein levels of JAK2-STAT3 and NF-κB signaling pathways in splenic CD4^+^ T cells on d 7 *in vivo*. As shown in Figure 6, CAP inhibition mediated by vagotomy-suppressed phosphorylation of JAK2 and STAT3 and enhanced phosphorylation of NF-κB p65 and IκB in splenic CD4^+^ T cells of VMC mice (*p*< 0.05). Pnu282987 treatment displayed the opposite effects in the VMC+Pnu group, reversing the effects of vagotomy in VMC mice (*p*< 0.05).

### *Protein expressions of JAK2-STAT3 and NF-κB signal pathways in cultured splenic CD4^+^ T cells* in vitro

**Figure 7.**
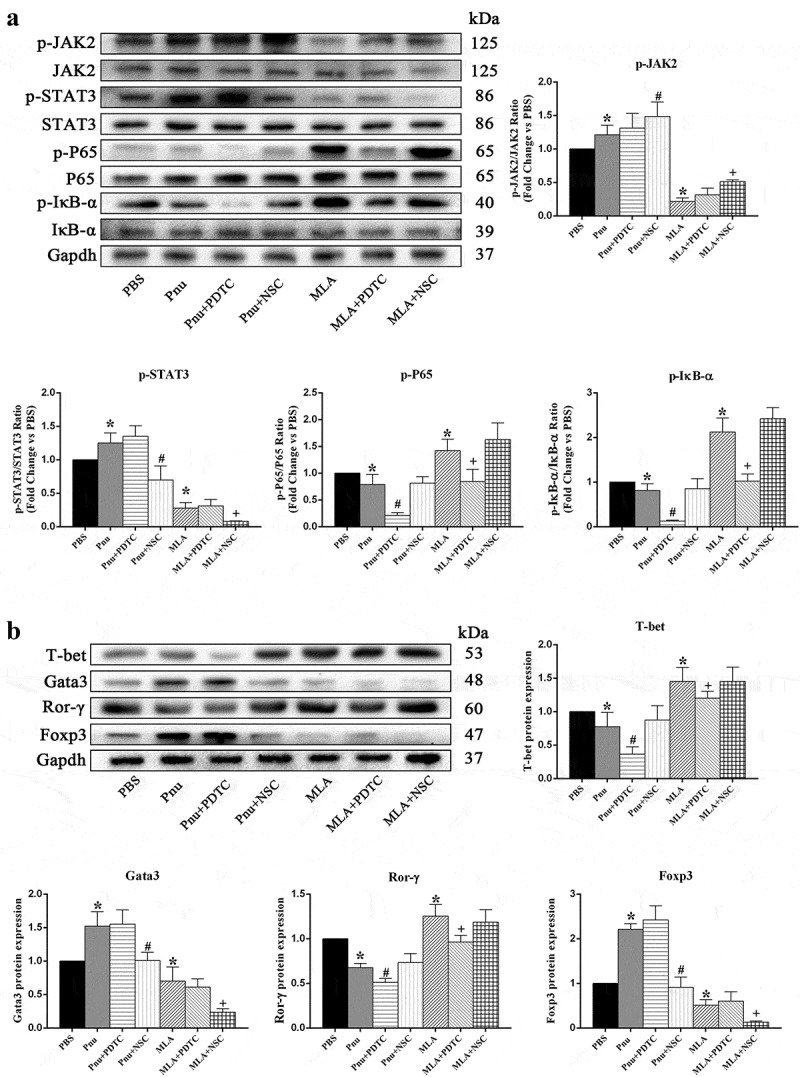
Western blot analysis demonstrating protein expression of JAK2-STAT3, NF-κB pathways, and specific transcription factors for Th cell subsets in cultured splenic CD4^**+**^ T cells on d 5 ***in vitro*** (*n* = 6 in each group). (a), Representative protein expression of signaling pathways and quantitative analysis of p-JAK2, p-STAT3, p-P65, and p-IκB-α expression in 7 groups of cells. (b), Representative protein expression of specific transcription factors and quantitative analysis of T-bet, Gata3, Ror-γ, and Foxp3 expression. Data are expressed as means ± SD. *p< 0.05 vs. PBS group. #*p*< 0.05 vs. Pnu group. +*p*< 0.05 vs. MLA group

In order to more accurately explore the mechanisms of Th cells differentiation, we isolated and cultured the splenic CD4^+^ T cells from CVB3-induced mice on d 7. The α7nAChR agonist Pnu and the α7nAChR antagonist MLA were administered to active and block CAP. MLA was used in vitro experiments instead of vagotomy. A selective NF-κB inhibitor PDTC and a STAT3 inhibitor NSC were used to detect the effect of signal pathways. According to the groups, CD4^+^ T cells were cultured with different treatments for next 5 d. As shown in Figure 7a, the phosphorylation protein expression ratios of p-JAK2/JAK2 and p-STAT3/STAT3 were decreased by 0.35- and 0.38-fold, and the phosphorylation ratios of p-P65/P65 and p-IκB-α/IκB-α were increased by 1.39- and 1.78-fold in MLA group compared with PBS group (*p*< 0.05). Pnu treatment upregulated the phosphorylation protein expression ratios of p-JAK2/JAK2 and p-STAT3/STAT3, and downregulated the phosphorylation ratios of p-P65/P65 and p-IκB-α/IκB-α in Pnu group compared with PBS group (*p*< 0.05). These results were similar with those *in vivo*. Furthermore, administering PDTC significantly reduced phosphorylation of NF-κB p65 and IκB, while administering NSC decreased phosphorylation of STAT3 and increased phosphorylation of JAK2 in Pnu or MLA group, respectively (Pnu+PDTC versus Pnu, MLA+PDTC versus MLA, Pnu+NSC versus Pnu, MLA+NSC versus MLA, *p*< 0.05).

### *The cultured splenic CD4^+^ T cell differentiation* in vitro

T-bet, Gata3, Ror-γ, and Foxp3 are specific transcription factors for Th1, Th2, Th17, and Treg cells, respectively. Thus, we detected the protein expression level of specific transcription factors in cultured splenic CD4^+^ T cells on d 5 *in vitro*. The protein expression of Gata3 and Foxp3 were increased by 1.54- and 1.69-fold, respectively, but the T-bet and Ror-γ expression were decreased by 0.73- and 0.68-fold in the Pnu group compared with the PBS group (*p*< 0.05). Nevertheless, MLA treatment downregulated the Gata3 and Foxp3 expression by 0.80- and 0.65-fold, respectively, while upregulating T-bet and Ror-γ expression by 1.62- and 1.44-fold in the MLA group compared with the PBS group (*p*< 0.05). Additionally, inhibiting NF-κB pathway through administering PDTC reduced the expression of T-bet and Ror-γ, whereas inhibiting STAT3 pathway through administering NSC decreased the expression of Gata3 and Foxp3 in the Pnu or MLA group, respectively (Pnu+PDTC versus Pnu, MLA+PDTC versus MLA, Pnu+NSC versus Pnu, MLA+NSC versus MLA, *p*< 0.05) (Figure 7b).

### *The percentage of cultured splenic CD4^+^ T cells* in vitro

**Figure 8.**
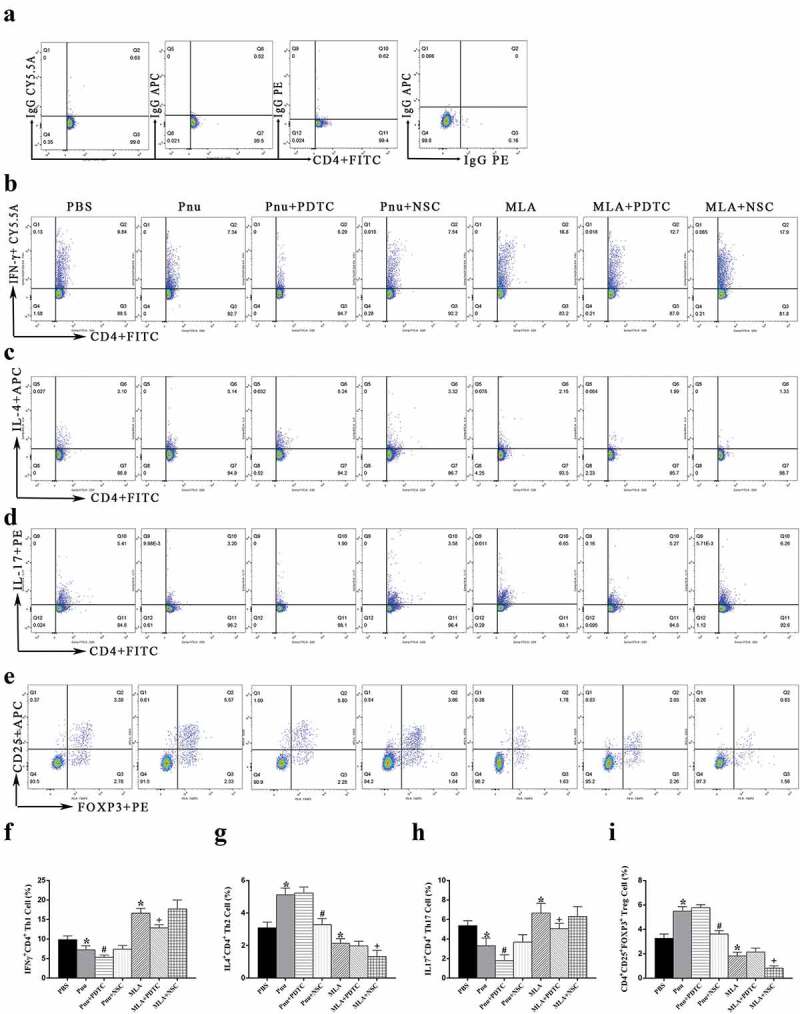
Percentages of Th subsets by flow cytometry analysis in cultured splenic CD4^+^ T cells on d 5 ***in vitro*** (*n* = 6 in each group). (a), Isotype controls for Th1, Th2, Th17, and Treg cells. (b) through (e), The representative results for each group. (f) through (i), The percentage of double-positive cells. Data are expressed as means ± SD. *p< 0.05 vs. PBS group. #*p*< 0.05 vs. Pnu group. +*p*< 0.05 vs. MLA group

On d 5, the percentage of Th subsets in cultured CD4^+^ T cells from CVB3-induced mice was analyzed. As shown in Figure 8, the percentages of Th1 and Th17 cells were lower in Pnu group and higher in MLA group than in PBS group, respectively (*p*< 0.05). Whereas, the percentages of Th2 and Treg cells were increased in Pnu group and decreased in MLA group compared with PBS group (*p*< 0.05). Inhibiting NF-κB pathway reduced the proportions of Th1 and Th17 cells, whereas inhibiting STAT3 pathway reduced the proportions of Th2 and Treg cells in the Pnu or MLA group, respectively (Pnu+PDTC versus Pnu, MLA+PDTC versus MLA, Pnu+NSC versus Pnu, MLA+NSC versus MLA, *p*< 0.05).

## Discussion

In the present study, we investigated the effects of the vagus nerve on VMC, demonstrating that right cervical vagotomy aggravates the inflammatory response through regulation of CD4^+^ T cell differentiation. We found that right cervical vagotomy increases the proportion of Th1 and Th17 cells, decreases the proportion of Th2 and Treg cells in the spleen, upregulates the expressions of T-bet, Ror-γ, IFN-γ, and IL-17, and downregulates expression levels of Gata3, Foxp3, and IL-4 in the heart. Furthermore, our study showed that blocking CAP inhibited JAK2-STAT3 activation, enhanced NF-κB activation in splenic CD4^+^ T cells, increased the percentages of Th1 and Th17 cells, and decreased the percentages of Th2 and Treg cells *in vitro*. Additionally, vagotomy-upregulated levels of proinflammatory cytokines, exacerbated the severity of myocardial lesions and cellular infiltration, and worsened cardiac function in VMC mice. In contrast, pnu282987 administration showed the opposite effects. Moreover, the abnormalities observed in VMC+Vag mice were alleviated by pnu282987 treatment. These results demonstrate that the vagus nerve plays a crucial role in protecting myocardium from virus infection, which may benefit by regulating CD4^+^ T cell differentiation in VMC. Activation of CAP by pnu282987 inhibited production of proinflammatory cytokines, regardless of the integrity of the vagus nerve in this model.

CAP mainly contains the vagus nerve, acetylcholine (ACh) secreted by the vagus nerve terminal, and α7nAChR from inflammatory cells [17]. The vagus nerve is a mixed nerve with an afferent fiber that makes up approximately 80% of the vagus nerve [26]. Previous studies have revealed that the afferent vagal fibers play a vital role in transferring peripheral inflammatory or pathogen-derived signals to the nucleus of the solitary tract. Additionally, it connects to the cranial nerve network in the central nervous system, and the efferent fibers carry parasympathetic motor control fibers that are related to communication in the autonomic nervous system from brainstem to organs [12]. These fibers release Ach, which then activates α7 nicotinic acetylcholine receptor (α7nAchR) on the surfaces of macrophages, lymphocytes, neurons, and other cells, exerting significant regulation of inflammatory cytokine production and inflammation [17]. The vagus nerve is the main component of a neural reflex mechanism (the inflammatory reflex) that controls innate immunity and inflammatory responses during pathogen invasions and tissue damage. The absence of this reflex (due to neural injury or absence of essential components) can lead to excessive innate immunity and cytokine toxicity [27]. Recently, studies have indicated that vagus nerve stimulation reduces the levels of proinflammatory cytokines in serum and local tissue, and vagotomy increases the levels of proinflammatory cytokines, aggravating inflammatory and immune lesions in experimental models of endotoxin, sepsis, ischemia/reperfusion, and other diseases [28]. Furthermore, vagotomy may affect the activation and recruitment of immune cells that participate in inflammation. Mihaylova et al. reported that vagotomy decreased immune cell counts in the spleen of rats with endotoxemia [29]. Therefore, activation of efferent vagus nerves could significantly regulate inflammatory and immune responses to improve diseases. In this study, CAP inhibition by unilateral cervical vagotomy increased proinflammatory cytokines and aggravated inflammation.

In recent decades, studies have shown that CAP induction by vagus nerve stimulation or an α7nAChR agonist reduces inflammation and improves disease states, and inhibiting CAP promotes inflammatory responses and aggravates diseases [30]. Additionally, investigators found that the spleen is vitally important to inhibit systemic inflammatory response by α7nicotinic agonists or vagus never stimulation. Splenectomy abolishes the reduction of inflammatory cytokines, regardless of stimulation of the efferent vagus nerve in sepsis and other inflammatory diseases [31]. In addition, the spleen is recognized as the largest immune organ, where CD4^+^ T cells play a crucial role in anti-inflammatory effects. Activating CAP with nicotine reduced the expression of Th17-related IL-17 and Ror-γ, increased expression of Th2-related IL-4 and Gata3, and attenuated the inflammatory response in a murine collagen-induced arthritis (CIA) model of rheumatoid arthritis [32]. Galitovskiv et al. found that nicotine reduced the inflammatory response by reducing the proportions of Th17 cells and increasing the numbers of Treg cells in ulcerative colitis [33]. In agreement with previous studies, we demonstrated that CAP regulation modifies the percentage of CD4^+^ T cells subsets in VMC. Flow cytometry results show that CAP inhibition from right cervical vagotomy increased the proportions of Th1 and Th17 cells and decreased the proportions of Th2 and Treg cells in the spleen. Furthermore, pnu282987 administration displayed opposite results, and the changes of Th cell subsets in VMC mice after vagotomy were alleviated by pnu282987 treatment. With the viral infection injured cardiomyocytes, the host immune response was activated, which led inflammatory immune cells to infiltrated and attacked myocardium. And CD4^+^ T cells and their cytokines played a vital role in mediating immune response during the development of VMC [34–36]. The results of Western blot and qPCR revealed that vagotomy operation could change the levels of Th cell-specific transcription factors and cytokines in myocardium. The expression levels of Th1 cell-related IFN-γ and T-bet as well as Th17 cell-related IL-17 and Ror-γ were upregulated. Th2 cell-associated IL-4 and Gata3 as well as Treg cell-associated Foxp3 expression levels were downregulated. These changes increased the inflammatory response, aggravated myocardial lesions, and worsened cardiac function in VMC+Vag mice.

The underlying mechanism regulating Th cell subset differentiation after activation or suppression of CAP is not well understood. We hypothesized that nuclear factor kappa B (NF-κB) [37] and signal transducer and activator of transcription (JAK-STAT) signaling pathways may participate in modulating the differentiation of CD4^+^ T cells. Wang et al. have confirmed that nicotine, α7nAChR agonist, could prevent the activation of NF-κB signaling pathway induced by endotoxin, and α7nAChR antagonist methllycaconitine has shown the opposite effect [38,39]. Nizri et al. found that nicotine, which activates the α7nAChR on the CD4^+^ T cells of autoimmune encephalomyelitis mice, significantly inhibited NF-κB signaling and prevented the overreaction of Th1 and Th17 cells, reducing inflammatory damage to the nervous system [40]. In myocarditis, left stellectomy reduced inflammation via activation of the JAK2-STAT3-mediated signaling cascade [41]. Mathur et al. found that STAT3 is required for Th17 cell differentiation [42]. De Jonge et al. proposed that activation of CAP by the α7nAChR agonist nicotine in macrophages incited JAK-STAT signaling, leading to the inhibition of NF-κB transcription factor, ultimately exerting an anti-inflammatory effect [43]. Increased STAT3 phosphorylation inhibits NF-κB DNA binding, which is probably involved in the direct interaction of dimerized STAT3 with the p65 subunits. These results indicate that the inhibitory effect of α7nAChR agonists or vagus nerve stimulation on NF-κB activation is modulated by protein–protein interactions. Our results indicated that CAP inhibition by vagotomy or MLA suppressed JAK2-STAT3 activation, enhanced NF-κB activation in splenic CD4^+^ T cells. Vagotomy or MLA treatment increased the proportion of Th1 and Th17 cells, and decreased the proportion of Th2 and Treg cells. Pnu administration reversed these changes. Inhibiting NF-κB pathway by PDTC reduced the percentages of Th1 and Th17 cells, whereas inhibiting STAT3 pathway by NSC reduced the percentages of Th2 and Treg cells in Pnu or MLA group, respectively. These results revealed that CAP regulates the differentiations of splenic CD4^+^ T cell subsets, related with JAK2-STAT3 and NF-κB signal transduction pathways.

However, the α7nAChR is expressed on the surface of CD4^+^ T cells and also on the surface of antigen-presenting cells (APCs). Machimo and colleagues’s reported (1) that α7nAChRs on APCs suppress CD4^+^ T cell activation; (2) that α7nAChRs on CD4^+^ T cells up-regulate the development of Tregs and effector T cells, most likely via activation of JAK2/STAT pathways [44]. Thus, α7nAChRs expressed by immune cells are crucially involved in the regulation of both innate and adoptive immunity. Moreover, the inhibitory effect on splenic macrophages by vagus nerve circuit is, at least in part, under the control of an independent non-neuronal lymphocytic cholinergic system (acetylcholine-producing T cells) [45,46]. Therefore, the contribution of acetylcholine-stimulated macrophages (or of their secreted cytokines) requires further study.

We measured levels of CVB3 RNA and proinflammatory cytokines in the six groups in the heart tissues. Vagotomy reduced the viral abundance in VMC on d 7, which might be related to activation of Th1 cells differentiation. Previous studies showed that Th1 cells inhibited viral replication but increase the systemic inflammatory response. Our study revealed that vagotomy increased the proportion of Th1 cells and IFN-γ, enhancing the anti-virus effect and systemic inflammatory response, which resulted in a net a deteriorating effect [8]. Additionally, the levels of CVB3 RNA, inflammatory cytokines, and Th cell-related transcription factors were not significantly different among the five myocarditis groups on d 14, which might be related to the progression of viral myocarditis. The virus is nearly cleared from the myocardium in VMC on d 14; at that time, it is progressing into the chronic phase [47].

## Conclusions

In our study, we preliminarily found that the vagus nerve plays a vital role in mediating anti-inflammatory effects via CAP-mediated regulation of CD4^+^ T cell differentiation in the early stage of VMC. Vagotomy increased the percentages of Th1 and Th17 cells and decreased the percentages of Th2 and Treg cells in the spleen. Those effects possibly changed the levels of specific transcription factors of CD4^+^ T cell subsets in the myocardium, increased the expression of pro-inflammatory mediators, expanded inflammatory infiltration and myocardial lesions in the acute and subacute phases of the viral myocarditis model. CD4^+^ T cell differentiation was related to JAK2-STAT3 and NF-κB signal transduction pathways. Additionally, cholinergic stimulation with pnu282987 treatment also exerted anti-inflammatory effects, regardless of the integrity of the vagus nerve. This study provides a new clue for understanding the role of vagus nerve during viral myocarditis progression, unveiling potential new target in the therapy against CVB3-induced myocarditis.

## Supplementary Material

Supplemental Material
