# Supplementary material for: Vagus nerve plays a pivotal role in CD4+ T cell differentiation during CVB3-induced murine acute myocarditis
Source: Virulence. 2021 Jan 15;12(1):360–76. doi: 10.1080/21505594.2020.1869384 (PMC7834089; doi:10.1080/21505594.2020.1869384)
Supplement: Supplemental Material [file KVIR_A_1869384_SM7573.docx]

**Table 1. Primer sequences used for molecular analysis**

| Gene (species) | 5’-3’ primer | 3’-5’ primer |
| --- | --- | --- |
| T-bet (mouse) | GCTCGGAACTCCGCTTCATAAC | CTGGACCCAACTGTCAACTGCTT |
| Gata3 (mouse) | GCCTCGACTTACATCCGAACCC | CCCATTACCACCTATCCGCCCTAT |
| Ror-γ (mouse) | CGACTTCCATTGCTCCTGCTTT | GCTGTGCCCACCACCTCACT |
| Foxp3 (mouse) | CTTGCGAAACTCAAATTCATCTACG | TCACCTATGCCACCCTTATCCG |
| CVB3 | GTCGGGCTTTCATTTGCTTA | CTGGTTGGGCACTCCTGTAT |
| GAPDH (mouse) | GGTTGTCTCCTGCGACTTCA | TGGTCCAGGGTTTCTTACTCC |

**Figure 1. Western blot analysis demonstrating protein expression of JAK2-STAT3, NF-κB pathways, and specific transcription factors for Th cell subsets in cultured splenic CD4^+^ T cells on day 5 in vitro.**

**
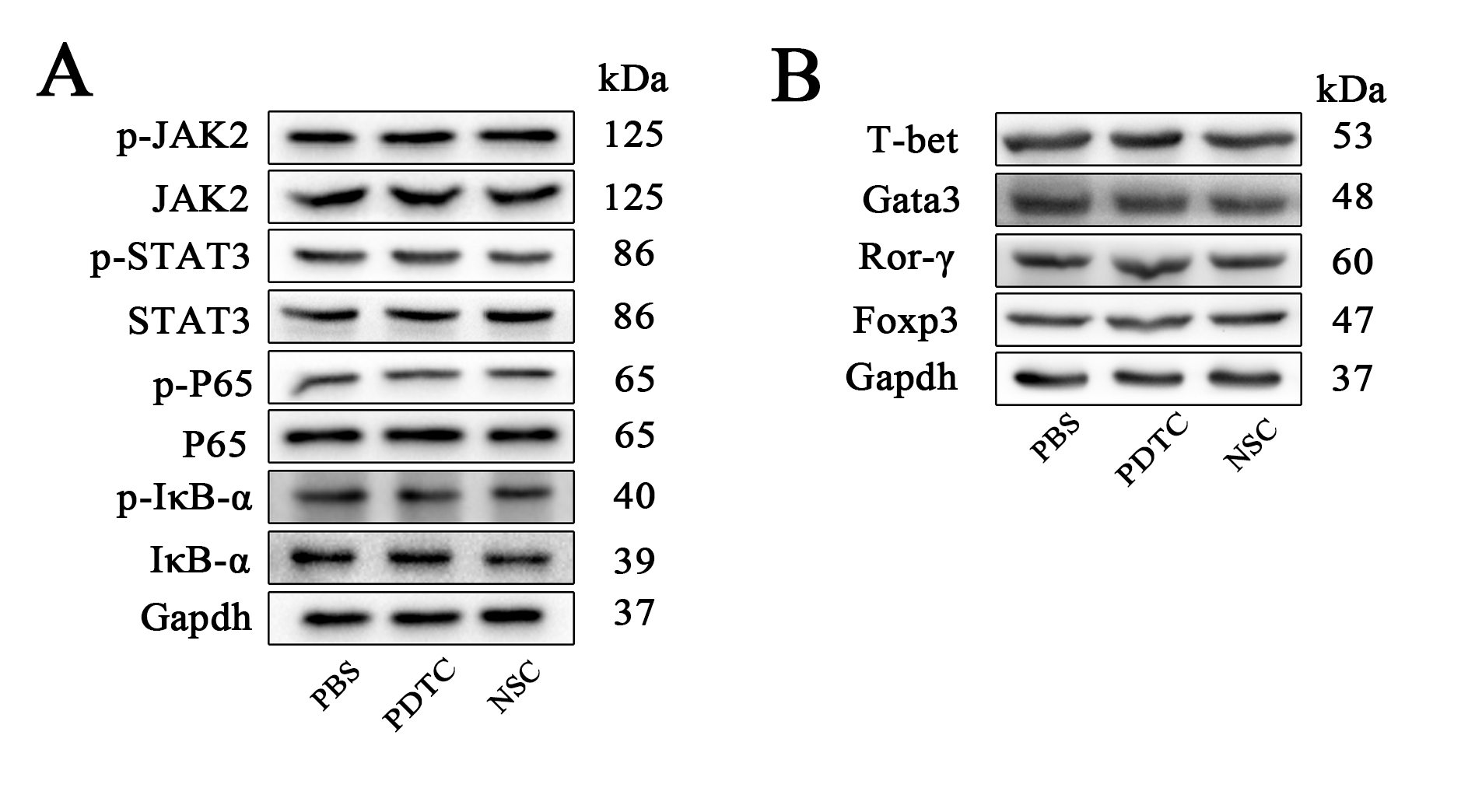
**
